# Sober living houses: research in northern and southern California

**DOI:** 10.1186/1940-0640-10-S1-A30

**Published:** 2015-02-20

**Authors:** Rachael A Korcha, Douglas L Polcin, Amy A Mericle, Jason Bond

**Affiliations:** 1 Alcohol Research Group, Emeryville, CA, 94608, USA

## Background

Sober living houses (SLHs) are peer-managed residences that require sobriety and household participation among residents who rent rooms on a monthly (indefinite) basis and otherwise live normal lives according to personal schedules and needs. The houses do not provide counseling or services, but regular attendance at 12-step or other types of mutual-help groups is generally required. Approximately 1000 sober living houses, members of two state-wide organizations, operate in California to serve a large and complex population. This presentation provides an overview of work conducted to date studying sober living houses in Northern and Southern California.

## Methods and results

In a study of SLHs in Northern California, 300 residents were followed for 18 months after entry. Our research found that neighbors and key informants (e.g., criminal justice, housing and political officials) were highly supportive of SLHs. Findings showed resident improvement in a variety of areas, including drug and alcohol use, employment, psychiatric symptoms, and arrests, with improvements that were maintained over the course of the study period. Although residents on parole and probation had substance use reductions that were comparable to voluntary residents, they had far more problems maintaining employment, higher rates of re-arrest and incarceration, and lower attendance to self-help groups. An ongoing randomized clinical trial in Southern California (anticipated N = 330 residents; 50 houses) is currently examining the effectiveness of an intervention to improve access to services and reduce HIV risk among sober living house residents on parole or probation. Residents randomized to the treatment condition receive a Motivational Interviewing Case Management (MICM) intervention specifically targeted to the problems presented for each resident. The list of potential problems that MICM can address is extensive and aims to help in a variety of problem areas inclusive of: 1) adapting to the sober living house environment; 2) complying with parole and probation; 3) finding and maintaining work; 4) successfully accessing and retaining services; 5) addressing HIV risk, testing, and treatment; 6) mobilizing personal and informal resources; and 7) managing setbacks (e.g., relapse, loss of housing, loss of work). Study participants are being tracked over a 12-month time period and being assessed on criminal justice, HIV risk, and drug and alcohol outcomes.

## Conclusions

Sober living houses play an important role in helping individuals in their recovery from substance abuse, and providing additional services in these houses (MICM) to increase access to formal services may further enhance outcomes for high-risk populations.

